# Parathyroidectomy Improves the Consumption of Erythropoiesis-Stimulating Agents in Hemodialysis Patients

**DOI:** 10.3390/ijms231810391

**Published:** 2022-09-08

**Authors:** Yu-Ting Lee, Chi-Wen Tu, Kam-Hong Kam, Tsung-Liang Ma, Chin-Ho Kuo, Ming-Yang Lee, Chih-Yen Hsiao, Michael W. Y. Chan, Peir-Haur Hung

**Affiliations:** 1 Division of Hematology and Oncology, Department of Medicine, Ditmanson Medical Foundation Chiayi Christian Hospital, Chiayi 60002, Taiwan; 2 Department of Biomedical Sciences, National Chung Cheng University, Min-Hsiung, Chiayi 621301, Taiwan; 3 Epigenomics and Human Disease Research Center, National Chung Cheng University, Min-Hsiung, Chiayi 621301, Taiwan; 4 Center for Innovative Research on Aging Society (CIRAS), National Chung Cheng University, Min-Hsiung, Chiayi 621301, Taiwan; 5 Division of General Surgery, Department of Surgery, Ditmanson Medical Foundation Chiayi Christian Hospital, Chiayi 60002, Taiwan; 6 Division of Thoracic Surgery, Department of Surgery, Ditmanson Medical Foundation Chiayi Christian Hospital, Chiayi 60002, Taiwan; 7 Division of Nephrology, Department of Medicine, Ditmanson Medical Foundation Chiayi Christian Hospital, Chiayi 60002, Taiwan; 8 Min-Hwei Junior College of Health Care Management, Tainan 73658, Taiwan; 9 Department of Hospital and Health Care Administration, Chia Nan University of Pharmacy and Science, Tainan 71710, Taiwan; 10 Department of Applied Life Science and Health, Chia Nan University of Pharmacy and Science, Tainan 71710, Taiwan

**Keywords:** end-stage renal disease, anemia, parathyroidectomy, secondary hyperparathyroidism, hemoglobin, TAST

## Abstract

Secondary hyperparathyroidism (SHPT) is common in end-stage renal disease (ESRD) patients, and it can suppress erythropoiesis. We aimed to investigate the relationship between the consumption of erythropoiesis-stimulating agents (ESAs) and parathyroidectomy (PTX) in ESRD patients with SHPT and to determine the predictors for anemia improvement. The current standard of chronic kidney disease anemia therapy relies on the prescription of iron supplementation, and ESA. We retrospectively analyzed 81 ESRD patients with PTX at Ditmanson Medical Foundation Chiayi Christian Hospital from July 2004 to Dec 2018. The requirement of ESA therapy markedly declined from a dose of 41.6 (interquartile range [IQR], 0–91.2) to 10.3 (IQR, 0–59.5, *p* = 0.001) unit/kg/week. In addition, 63.7% of patients required iron replacement therapy preoperatively and the proportion reduced to 52.5% after PTX (*p* < 0.001). The hemoglobin (Hb) level showed an insignificant change from a median value of 10.7 g/dL (9.5–11.6 g/dL) before PTX to 10.5 g/dL (9.6–11.2 g/dL) at 6 months after PTX. A preoperative Hb level ≤ 10 mg/dL (odds ratio [OR], 20.1; 95% confidence interval [CI], 4.71–125, *p* < 0.001) and transferrin saturation (TSAT) < 25% (OR, 12.8; 95% CI, 2.51–129, *p* < 0.001) were predictors for anemia improvement. Our study demonstrated that PTX markedly decreased the requirement of ESA. Patients with a low preoperative Hb level or low TSAT showed an increase in the Hb level after PTX. PTX may be considered not only for SHPT with refractory anemia but also for high ESA-dependent patients.

## 1. Introduction

Secondary hyperparathyroidism (SHPT), a common complication of end-stage renal disease (ESRD), results from abnormal changes affecting calcium homeostasis and presents with the increased secretion of parathyroid hormone (PTH) [1]. SHPT is associated with renal osteodystrophy, cardiovascular calcification, immune dysfunction, anemia, and erythropoiesis-stimulating agent (ESA) resistance [2,3,4]. SHPT is currently treated using phosphate binders, calcimimetics, calcitriol, or vitamin D analogs. Parathyroidectomy (PTX) is a surgical treatment to remove the parathyroid glands that secret PTH. PTX is recommended for severe SHPT patients who fail to respond to medical or pharmacological therapy [5,6].

Traditionally, iron supplementation and ESAs are current treatment options for correcting anemia and avoiding the need for red cell transfusion [7]. All ESAs effectively increase hemoglobin (Hb) levels in a substantial percentage of patients. However, the use of ESAs has been surrounded by safety issues in increased cardiovascular risk, especially when used at high doses in inflamed and hyporesponsive patients [8]. Furthermore, exposure to ESAs may also induce autoantibodies against erythropoietin receptors leading to ESA resistance [9]. Considering the adverse effects of ESAs, new strategies for correcting anemia are awaited.

PTX has been reported to be associated with anemia. Excessive secretion of PTH can cause bone marrow fibrosis, inhibit endogenous erythropoietin (EPO), suppress erythroid progenitors, and reduce red blood cell survival [3,10]. Thus, treatment of SHPT is believed to improve anemia and decrease the requirement for ESA treatment [11]. Because PTX can reduce the dose of ESA, it can thereby potentiate the effect of ESA. PTX has been reported to improve anemia and reduce the dose of ESA in patients with SHPT, but these data were not consistent [12,13,14]. In addition, our previous study suggested that chronic cytopenia was a consequence of long-term hemodialysis but PTX did not reverse or affect the patients’ hematologic profile [15]. We proposed that this insignificance could be attributed to heterogeneous entities in ESRD. Furthermore, the associated factors used to predict postoperative improvement in anemia are still not known. Therefore, we conducted this retrospective study to investigate the consumption of ESA with PTX and to identify possible factors for anemia improvement.

## 2. Results

### 2.1. Clinical Characteristics of the Study Population

**Figure 1:**
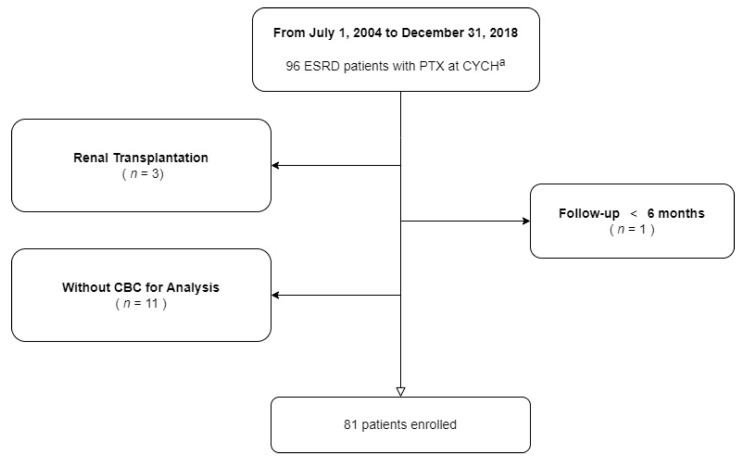
Selection flowchart ^a^ Chiayi Christian Hospital.

Overall, 96 ESRD patients receiving PTX were registered at the Ditmanson Medical Foundation Chiayi Christian Hospital at the end of our study. Three post-renal transplantation patients, one patient who underwent follow-up for less than 6 months, and 11 patients with limited evaluated data were excluded from our cohort (Figure 1, Table S1). Ultimately, 81 patients (40 male and 41 female) with a follow-up period of 13.1 years (interquartile range [IQR], 9.9–6.5 years) were enrolled in this study. The majority of our patients (67%) received ESA therapy. The median age at ESRD was 46.8 years (37.3–56.7 years) and that at PTX was 53.1 years (47.9–63.5 years). The median time from ESRD to the first PTX was 5.7 years (3.7–9.7 years).

### 2.2. Changes in the Hematological Profile, ESA Doseage, and Iron Replacement Therapy after Parathyroidectomy

**Figure 2:**
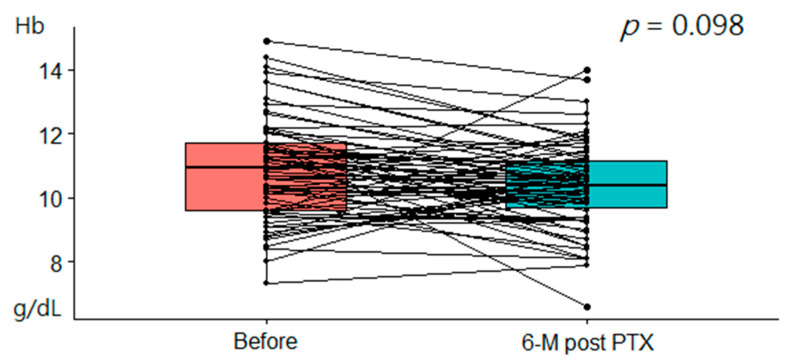
Comparison of Hb levels at baseline and 6 months post-PTX.

The white blood cell (WBC) count, Hb level, and platelet count did not change significantly after surgery. The Hb level slightly decreased from 10.7 g/dL (IQR, 9.5–11.6 g/dL) preoperatively to 10.5 g/dL (IQR, 9.6–11.2 g/dL, *p* = 0.098) at 6 months post-PTX (Figure 2) and 10.5 g/dL (IQR, 9.5–11.1 g/dL, *p* = 0.109) at 12 months post-PTX.

**Figure 3:**
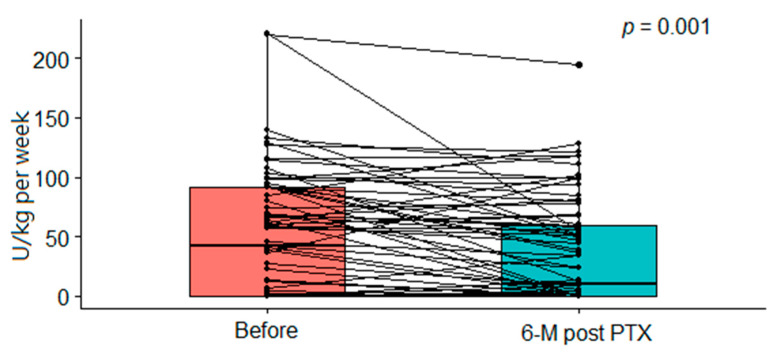
Comparison of accumulative ESA dosage at baseline and 6 months post-PTX.

The requirement of ESA therapy markedly declined from a dose of 41.6 (IQR, 0–91.2) unit/kg/week to 10.3 (IQR, 0–59.5, *p* = 0.001) unit/kg/week (Figure 3). Furthermore, 63.7% of patients required iron replacement therapy preoperatively and the proportion reduced to 52.5% after PTX (*p* < 0.001). The patients’ clinical characteristics are summarized in Table 1.

### 2.3. Factors Associated with Anemia Improvement

We used a logistic regression model to assess the associated factors. In the analysis, baseline Hb level ≤ 10 mg/dL (OR, 20.1; 95% CI, 4.71–125, *p* < 0.001), Ca level < 9.5 mg/dL (OR, 4.28; 95% CI, 1.25–15.2, *p* = 0.009), and transferrin saturation (TSAT) < 25% (OR, 12.8; 95% CI, 2.51–129, *p* < 0.001) were associated with anemia improvement (Table 2). Sex, age, and comorbidities did not show significance.

**Figure 4:**
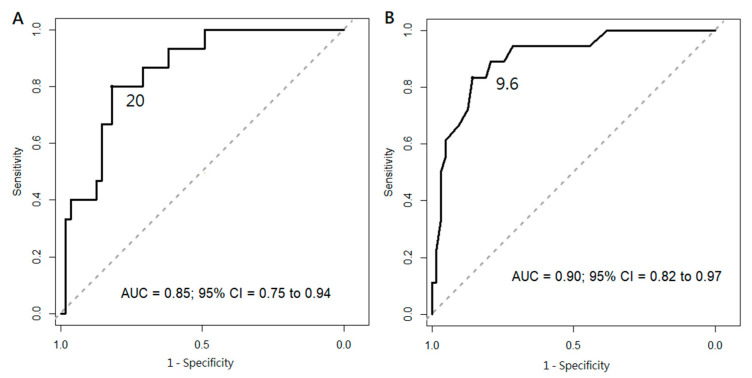
Receiver operating characteristic curve analysis. (**A**) Preoperative TSAT, cutoff value at 20%, sensitivity: 81.8%, specificity: 80% (**B**) Preoperative Hb, cutoff value at 9.6 g/dL, sensitivity: 85.7%, specificity: 83.3%.

Receiver operating character (ROC) analysis was performed to determine the predictive value of preoperative TSAT and Hb in detecting anemia improvement (Figure 4). A cutoff TSAT value of 20% showed 80% sensitivity and 81.8% specificity with an area under the curve (AUC) of 0.851. On the other hand, a preoperative Hb level of 9.6 g/dL showed optimal sensitivity (83.3%) and specificity (85.7%) for predicting anemia improvement (AUC = 0.902).

### 2.4. Analysis of Outcomes of Patients after PTX

**Figure 5:**
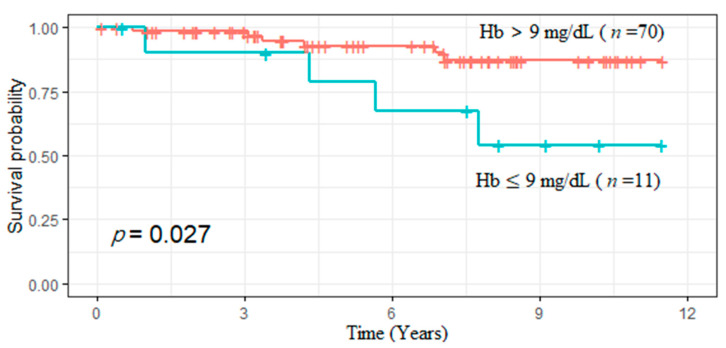
Preoperative hemoglobin ≤ 9 mg/dL was associated with poor survival.

In our cohort, 10 patients died at the end of the follow-up period. The cause of mortality included lung cancer (*n* = 1), pancreatitis (*n* = 1), sepsis (*n* = 3), cardiovascular disease (*n* = 1), and out-of-hospital cardiac arrest (*n* = 4). Interestingly, we found that patients with preoperative hemoglobin ≤ 9 mg/dL had poorer survival (HR, 3.76; 95% CI, 1.06–13.4, *p* = 0.04). Figure 5 shows the survival curves in different groups (*p* = 0.027 for the log-rank test). In addition, 7 of the 81 patients (14%) had recurrent hyperparathyroidism and underwent re-exploration for hyperthyroidism. No relationship was found.

## 3. Discussion

In the present study, we demonstrate the ESA dosing declined after PTX. Generally, PTX did not alter patients’ hematological profiles, including white blood count, hemoglobin level, and platelet count. We observed that patients with hemoglobin level ≤ 10 mg/dL and TSAT < 25% tend to show improvement in anemia (>0.5 g/dL) after PTX.

Several studies have demonstrated that PTX improves renal anemia in patients with SHPT (Table 3). For example, Mandolfo et al. reported a significant increase in the Hb level after PTX, regardless of whether ESA treatment was administered (8.6 to 10.4 g/dL) or not administered (10.0 to 11.1 g/dL). The consumption of the ESA dose significantly decreased from 170 to 96 units/kg per week after 6 months [12]. A similar finding was also observed by Yasunaga et al. They reviewed 29 patients and noticed a transient decline in the Hb level at 2 weeks after PTX but a subsequent increase at 3 months (10.2 to 11.2 g/dL) [13]. Chow and coworkers showed that anemia improved (8.6 to 9.4 g/dL) after PTX. In contrast to the findings reported by Mandolfo et al., Chow et al. found that the benefit was limited to patients who received ESA therapy. However, they did not show if PTX affected ESA treatment [16]. In contrast, Trunzo et al. retrospectively analyzed 37 patients and reported that PTX decreased the EPO dose requirement from 10,086 ± 1721 to 3514 ± 620 units/week and caused an insignificant upward trend in Hb levels (11.4 to 12.1 g/dL) [14]. Our data demonstrated no significant change in hemoglobin levels after PTX, but PTX would reduce the dosage of ESA therapy. ESA therapy effectively increases Hb levels and improves patients’ quality of life. Nevertheless, more and more data suggested higher Hb concentrations increase the risk of ischemic stroke among dialysis patients [17]. In addition to Hb level, some types of ESA may be associated with mortality. We supposed that patients with PTX might benefit from the reduction of ESA consumption [18].

We consider that the severity of preoperative anemia is an important factor. Here, we found that patients with preoperative hemoglobin levels ≤ 10 mg/dL can potentially show an improvement in anemia (OR, 20.1; 95% CI, 4.71–125, *p* < 0.001). In comparison with our cohort and the cohort evaluated by Trunzo et al. [14], the populations evaluated by Mandolfo et al. [12], Yasunaga et al. [13], and Chow et al. [16] had a relatively lower preoperative Hb level, which probably led to a significant result. In addition, Dr. Jemcov reported a case series with nine patients receiving PTX. Among them, five patients with preoperative hemoglobin levels ≤ 10 mg/dL showed anemia improvement after PTX [19].

The effect of PTX on erythropoiesis has been identified. First, Rao et al. [10] established a correlation between the extent of bone marrow fibrosis and the severity of SHPT. PTX reverses the bone marrow fibrosis caused by SHPT and improves cytopenia [23]. Another proven mechanism is the intrinsic EPO production that begins immediately after PTX and continues up to 12 months. Furthermore, the increase in EPO is for up to approximately five times the baseline value [13,24].

Our data also demonstrated marked improvement of TSAT in the anemia improvement group. Functional iron deficiency anemia (IDA), also known as iron-restricted erythropoiesis with a poor ability to recruit iron from storage for erythropoiesis, is usually diagnosed with low TSAT but a variable ferritin level (usually < 800 ng/dL) [25,26]. We noticed the proportion of cases showing functional IDA conspicuously decreased (20% to 9.7 %, *p* = 0.003) after PTX. PTH is known to stimulate hepatocytes to produce interleukin-6 (IL-6) [27]. IL-6 plays an important role in iron homeostasis by induction of hepcidin and promotion of iron acquisition into macrophages [28,29]. Apart from the reversal of bone marrow fibrosis and enhanced erythropoietin production [13,23], we propose that the removal of excess PTH also contributed to improvement in iron-restricted erythropoiesis.

Finally, some articles have reported that PTX is associated with reduced mortality in hemodialysis patients with SHPT [18,30]. Successful PTX may reduce the risk of all-cause and cardiovascular mortality in hemodialysis patients with severe, uncontrolled SHPT [30]. Here, we also observed that patients with preoperative low Hb levels had a worse outcome. Although our analysis had limited cases, lower Hb levels and higher PTH levels preoperatively might reflect a serious SHPT resulting in more fatal cardiovascular disease events. A meta-analysis conducted by van Ballegooijen, A. J., et al. revealed a positive association between PTH excess and cardiovascular events [31,32]. For example, cardiovascular calcification in these patients and decreased renal function are known to be independent risk factors for cardiovascular disease. In CKD patients undergoing hemodialysis, cardiovascular events are responsible for nearly 50% of mortality, and the presence and extent of vascular calcification are strong predictors of cardiovascular mortality in these patients. Patients with cardiovascular disease are also more prone to PTH excess and have a higher risk of secondary cardiovascular events [31].

This study had several limitations. First, this was a small, single-center retrospectively designed cohort, so selection bias was inevitable. For example, patients who refused PTX were elderly and fragile and were not enrolled [15,18]. Second, we lacked bone marrow and cytogenetic data to measure the degree of bone marrow fibrosis and examine myelodysplastic syndrome, a hematopoietic disease. The cumulative incidence of myelodysplastic syndrome increases with the duration of dialysis [33]. Lastly, data for some factors, such as prior medical treatment for SHPT, were not available for analysis. Thus, further prospective studies are needed to validate our findings.

## 4. Materials and Methods

### 4.1. Study Population

We retrospectively reviewed the data for ESRD patients with SHPT who were treated with PTX at Ditmanson Medical Foundation Chiayi Christian Hospital from July 2004 to December 2018. Patients who underwent renal transplantation or were followed up for less than 6 months were excluded. All patients were observed until loss of follow-up, death, or 31 December 2018, whichever was earlier. Participants’ clinical information (including age, sex, body mass index [BMI]) and information related to comorbidities (including hepatitis C virus [HCV], hepatitis B virus [HBV], chronic liver disease, rheumatologic disease, diabetes mellitus [DM], cerebral vascular disease, hypertension, and cancer) were obtained as baseline characteristics for further analysis. This retrospective study was duly approved by the institutional review board of Ditmanson Medical Foundation Chiayi Christian Hospital (CYCH-IRB-2021055).

### 4.2. ESA, Hematological and Biochemical Measurements

For ESRD patients, laboratory data, including complete blood count (CBC), calcium, phosphate, albumin, and uric acid levels, the calcium × phosphate product (Ca × P product), and alkaline phosphatase level were examined at least every month or as clinically indicated. Ferritin and TSAT were assessed every 3 months, and the intact parathyroid hormone (iPTH) level was measured biannually. We examined the difference in the Hb levels at baseline and 6 months post-PTX. To minimize measurement error, we determined that anemia improvement indicated that Hb levels raised more than 0.5 g/dL at 6 months post-PTX (Hb at 6 months post-PTX subtract Hb at baseline). Additional causes of mortality were reviewed in our cohort.

ESA was treated initially at 20 to 50 IU/kg/week when patients had Hb < 9g/dL and the dosage of ESA might be subsequently increased to maintain hemoglobin level but not exceed 11 g/dL. The maximal dose of ESA was capped at 5000 U per week due to Taiwan’s National Health Insurance Reimbursement Policy [17,34]. The cumulative dosage of ESA 6 months before PTX and 6 months after PTX were calculated and compared. Here, we defined a dose conversion ratio of 200 U of epoetin to 1 mg of darbepoetin alfa [35].

Patients’ CBC was calculated by automated hematology analyzers (XE-5000, Sysmex Corp., Kobe, Japan). Patients’ whole blood was collected with EDTA anticoagulant, and the samples were analyzed within 4 h of collection. Reagents are specialized reagents for Sysmex instruments according to the manufacturer’s protocol. The iPTH level was determined using a chemiluminescence immunoassay (CLIA; Immulite 2000) [36]. Chronic liver disease was defined as a persistent inflammatory condition of the liver in which biochemical and imaging abnormalities were present over 6 months. The following disorders were described as rheumatic diseases: rheumatoid arthritis, systemic lupus erythematosus, Sjogren’s syndrome, and spondyloarthropathies. Functional iron deficiency was defined as a TSAT < 20% and a ferritin level between 100 and 800 ng/mL [26].

### 4.3. Statistical Analysis

The baseline characteristics of the enrolled patients in our cohort were displayed as the total number (*n*) and proportion (%). Factors with a normal distribution were expressed as mean ± standard deviation. Parameters without a normal distribution were presented as median and interquartile ranges. We used a generalized estimating equation model to access the correlation of longitudinal and repeatedly measured outcomes. We used logistic regression models to assess the association between anemia improvement and various factors. Risk factors with *p* < 0.1 in the univariate model were selected for further evaluation in the multivariate analysis. ROC curves and AUC were calculated. Post-PTX survival was measured as the date of the first PTX to the date of death or the last follow-up visit, and the survival curve was illustrated using the Kaplan–Meier method. Data management and statistical analysis were conducted using R software, version 4.1.0 (R Foundation for Statistical Computing, Vienna, Austria).

## 5. Conclusions

Our cohort indicated that PTX may improve ESA resistance and contribute to a prominent anemia improvement among patients with low hemoglobin and low TSAT at baseline. The improvement in Hb may be associated with iron-restricted erythropoiesis. Despite new drug development, PTX remains an effective strategy to control SHPT [6]. In addition to bone loss and vascular calcification, PTX may be also considered for patients with refractory anemia or intolerance to high ESA therapy.

## Figures and Tables

**Table 1:** Characteristics of patients with parathyroidectomy (*n* = 81).

Characteristics	Total (*n* = 81)		
	*n* (%), Median (IQR)		
Gender			
Female	40 (49.4)		
Male	41 (50.6)		
Median age at HD, years	46.8 (37.3–56.7)		
Median age at PTX, years	53.1 (47.9–63.2)		
Duration of follow-up, years (IQR)	13.1 (9.9–16.5)		
Time from 1st HD to PTX, years (IQR)	5.7(3.7–9.7)		
BMI (kg/m^2^)	23.2(20.5–26.8)		
Comorbidities			
HCV	10 (13.6)		
HBV	11 (14.8)		
Liver disease	12 (16)		
Rheumatologic disease	8 (10.6)		
Diabetes mellitus	27 (35.5)		
Cerebral vascular disease	10 (13.1)		
Hypertension	63 (82.8)		
Cancer	5(20.2)		
	Pre-PTX (IQR)	6 months Post-PTX (IQR)	* *p*
Hemoglobin (g/dL)	10.7 (9.5–11.6)	10.5 (9.6–11.2)	0.062
ESA dosage (Unit/Kg/week)	41.6 (0–91.2)	10.3 (0–59.5)	0.008
iP (mg/dL)	6.0 (4.9–7.2)	3.7 (2.7–4.8)	<0.001
Ca (mg/dL)	9.8 (9.3–10.2)	8.2 (7.0–9.2)	<0.001
Ca × P	58.2(48.0–71.0)	30.1 (20.2–40.0)	<0.001
Ferritin (ng/mL)	395 (249–572)	485 (287–630)	0.284
TSAT (%)	25.9 (18.4–37.0)	28.4 (22.4–43.1)	0.015
PTH (pg/mL)	1807(1383–2346)	152 (68.6–340)	<0.001
Functional IDA	14 (20%)	7 (9.7%)	0.003
Iron replacement therapy	51 (63.7%)	42 (52.5%)	0.001

BMI, Body mass index; ESA, Erythropoietin stimulating agents; HBV, Hepatitis B; HCV, Hepatitis C; HD, Hemodialysis; IQR, interquartile range; IDA, Iron deficiency anemia; iP, inorganic phosphorus; Ca, calcium; Ca × P, the product of calcium and phosphorus; PTH, parathyroid hormone; PTX, parathyroidectomy; TAST, transferrin saturation. * Analysis based on generalized estimating equations; adjusted for gender, BMI, age, liver disease, hematologic disease, diabetes mellitus, cerebral vascular disease, and hypertension.

**Table 2:** Factors for Anemia Improvement.

Predictive Variables	OR (95% CI)	*p* Value
Age ≤ 45 (at HD)	2.36 (0.61–10.2)	0.231
Age ≤ 55 (at PTX)	3.13 (0.85–14.5)	0.063
Age at PTX	0.97 (0.92–1.01)	0.175
Male Gender	1.37 (0.47–3.94)	0.553
Hb ≤ 10 mg/dL	20.1 (4.71–125)	<0.001
iP ≥ 5.5	1.69 (0.49–6.86)	0.417
Ca < 9.5 mg/dL	4.28 (1.25–15.2)	0.009
Ca × P > 55	0.75 (0.22–2.46)	0.603
Ferritin > 500 mg/dL	0.79 (0.19–2.86)	0.777
TSAT < 25%	12.8 (2.51–129)	<0.001

OR, odd ratio; CI, confidence interval; Hb, hemoglobin; HD, hemodialysis; iP, inorganic phosphorus; Ca, calcium; Ca × P, the product of calcium and phosphorus; PTX, parathyroidectomy; TAST, transferrin saturation.

**Table 3:** Studies demonstrated that PTX improve renal anemia in patients with SHPT.

Study	N	Result
**Positive association with Hb**		
Mandolfo, S. et al., (1998) [12]	39	Two months post-PTX ESA treatment: 8.6 ± 1.0 to 10.4 ± 1.2 g/dL (*p* < 0.005) Non-ESA treatment: 10 ± 1.3 to 11.1 ± 1.1 g/dL (*p* < 0.01) ESA use: 170 ± 67 → 96 ± 78 U/Kg/wk after 6 months (*p* < 0.005)
Yasunaga, C. et al., (2002) [13]	29	6 months post-PTX: 10.2 ± 1.5 to 11.2 ± 1.2 g/dL (*p* < 0.001) ESA use: 2304 →2304 U/wk at 6 months (insignificant)
Chow, T.L. et al., (2007) [16]	23	6 months post-PTX: 8.6 ± 2.1 vs 9.4 ± 2.1 g/dL (*p* = 0.04)
Sharma, J. et a, (2012) [19]	150	Increases in Hematocrit: 32.6 ± 5.4% to 35.4 ± 4.8% (*p* < 0.001)
Chen, C. et al., (2015) [20]	16	6 months post-PTX: 8.8 ± 9.1 to 11.4 ± 1.4 g/dL (*p* < 0.01) ESA use: 160.81 ± 13.53 → 124.96 ± 26.41 U/Kg/wk (*p* < 0.05)
Gong, W. et al., (2021) [21]	87	6 months post-PTX: 9.2 ± 1.4 to 12.6 ± 1.2 g/dL (*p* < 0.05)
**No association with Hb**		
Trunzo, J.A. et al., (2008) [14]	37	12 months post-PTX: 11.4 ± 0.3 to 12.1 ± 0.2 g/dL (insignificant) ESA use:10,086 ± 1721 to 3514 ± 620 U/week (*p* < 0.05)
Jemcov, T.K. et al., (2008) [22]	9	6 months post-PTX: 8.8 ± 1.9 to 9.9 ± 1.0 g/dL (insignificant)

## Data Availability

The data presented in this study are available on request from the corresponding author.

## Supplementary Materials

The following supporting information can be downloaded at: https://www.mdpi.com/article/10.3390/ijms231810391/s1.
