# Supplementary material for: Parathyroidectomy Improves the Consumption of Erythropoiesis-Stimulating Agents in Hemodialysis Patients
Source: Int J Mol Sci. 2022 Sep 8;23(18):10391. doi: 10.3390/ijms231810391 (PMC9499136; doi:10.3390/ijms231810391)
Supplement: Supplementary file 1 [file ijms-23-10391-s001.zip › ijms-1875479-SI.pdf]

Table S1 The effects of parathyroidectomy on ESA therapy

Parameter	β	SE	Wald	p-value
Intercept	-13.7	34.6	0.15	0.692
ESA therapy				
Pre-PTX vs Post-PTX	-10.6	4.03	6.97	0.008
Sex (male)	-10.4	11.3	0.83	0.361
Age at HD	0.59	0.48	1.46	0.227
BMI	1.47	1.08	1.85	0.173
Functional IDA	16.5	10.9	2.28	0.131
Comorbidities				
Liver disease	-29.1	13.7	4.47	0.034
Rheumatologic disease	18.2	23.1	0.62	0.430
Diabetes mellitus	-6.73	11.1	0.47	0.490
Cerebral vascular disease	-27.8	12.2	5.19	0.023
Hypertension	6.25	15.5	0.16	0.687
